# Patency of conduits in patients who received internal mammary artery, radial artery and saphenous vein grafts

**DOI:** 10.1186/s12872-020-01433-0

**Published:** 2020-03-24

**Authors:** Alistair Royse, William Pamment, Zulfayandi Pawanis, Sandy Clarke-Errey, David Eccleston, Andrew Ajani, William Wilson, David Canty, Colin Royse

**Affiliations:** 1 Department of Surgery, The University of Melbourne, PO Box 2135 RMH, Melbourne, 3050 Australia; 2 Department of Cardiothoracic Surgery, The Royal Melbourne Hospital, PO Box 2135, Melbourne, Victoria 3050 Australia; 3 Universitas Airlangga Hospital, Universitas Airlangga, Surabaya, Indonesia; 4 Statistical Consulting Centre, The University of Melbourne, 139 Barry St, Parkville, 3010 Australia; 5 Department of Medicine and Cardiology, Royal Melbourne Hospital, The University of Melbourne, Melbourne, Australia; 6 Department of Medicine, Monash University, Clayton, Australia; 7 Department of Anaesthesia and Pain Management, The Royal Melbourne Hospital, Melbourne, Australia

**Keywords:** (Max 10) patency, Arterial, Saphenous vein, Radial artery, Internal mammary artery

## Abstract

**Background:**

Where each patient has all three conduits of internal mammary artery (IMA), saphenous vein graft (SVG) and radial artery (RA), most confounders affecting comparison between conduits can be mitigated. Additionally, since SVG progressively fails over time, restricting patient angiography to the late period only can mitigate against early SVG patency that may have occluded in the late period.

**Methods:**

Research protocol driven conventional angiography was performed for patients with at least one of each conduit of IMA, RA and SVG and a minimum of 7 years postoperative. The primary analysis was perfect patency and secondary analysis was overall patency including angiographic evidence of conduit lumen irregularity from conduit atheroma. Multivariable generalized linear mixed model (GLMM) was used. Patency excluded occluded or “string sign” conduits. Perfect patency was present in patent grafts if there was no lumen irregularity.

**Results:**

Fifty patients underwent coronary angiography at overall duration postoperative 13.1 ± 2.9, and age 74.3 ± 7.0 years. Of 196 anastomoses, IMA 62, RA 77 and SVG 57. Most IMA were to the left anterior descending territory and most RA and SVG were to the circumflex and right coronary territories. Perfect patency RA 92.2% was not different to IMA 96.8%, *P* = 0.309; and both were significantly better than SVG 17.5%, *P* < 0.001. Patency RA 93.5% was also not different to IMA 96.8%, *P* = 0.169, and both arterial conduits were significantly higher than SVG 82.5%, *P* = 0.029. Grafting according to coronary territory was not significant for perfect patency, *P* = 0.997 and patency *P* = 0.289. Coronary stenosis predicted perfect patency for RA only, *P* = 0.030 and for patency, RA, *P* = 0.007, and SVG, *P* = 0.032.

When both arterial conduits were combined, perfect patency, *P* < 0.001, and patency, *P* = 0.017, were superior to SVG.

**Conclusions:**

All but one patent internal mammary artery or radial artery grafts had perfect patency and had superior perfect patency and overall patency compared to saphenous vein grafts.

## Background

Many confounding factors interact to limit direct comparisons between coronary bypass conduits, including coronary target grafting preferences, techniques of reconstruction, medications, individual patient differences or duration postoperatively. For an individual patient, some factors such as age, gender, medications or comorbid diseases should affect conduits equally. For group comparisons of conduits, if each patient has all three of the comparison conduits, this reduces bias compared to some patients not receiving one or more of the conduits.

Our institutional practice had relatively few patients that received all three conduits of internal mammary artery (IMA), radial artery (RA) and saphenous vein (SVG) at the same time, with subsequent experience being predominantly total arterial revascularization [1, 2]. We wished to examine conduit patency in the late postoperative period without the confounding influence of conduits that may exhibit early patency but could later have occluded, as is known to be the case with SVG where patency at 10 years is 47–64% [3–9]. Alternatively, arterial conduits may fail in the early period thought to relate to flow competition from the native coronary circulation but with little evidence of progressive failure over the mid or late postoperative periods. Grafting strategies are usually biased where the left IMA (LIMA) is generally anastomosed to the left anterior descending artery (LAD) which has the highest patency; whereas other conduits are used to revascularize the right coronary artery (RCA) which has the lowest patency [1, 10]. Stenosis or occlusion of the conduit may lead to recurrence of angina, myocardial infarction or heart failure, with reduced survival. We previously found that there was reduced survival even with the use of a single SVG as well as for multiple SVG conduits when compared to total arterial revascularization [11].

The angiographic classification of Fitzgibbon appears relevant only to SVG, since in our observations late angiography of arterial conduits appear to exhibit entirely normal lumen appearance; or are occluded [12]. We hypothesize that if an arterial conduit appears angiographically normal after 10 years postoperative, then it is unlikely to ever fail. Alternatively, if SVG appears irregular, but patent, after 10 years, then it would be expected that some progression of the conduit atherosclerosis would occur which may lead to eventual occlusion of the conduit. We therefore did not use the Fitzgibbon classification.

The presence or absence of symptoms may bias angiographic patency; and survival in the late period could bias results by selecting only long-term survivors. However, *within each individual patient*, many of the confounding variables can be eliminated if each patient had all three conduits of interest used.

The primary analysis was perfect patency where the presence of conduit lumen irregularity (atheroma) is absent, which may predict ongoing long-term preservation of patency. The secondary analysis was absolute patency where the presence of conduit lumen irregularity may predict possible ongoing loss of conduit patency in the long-term.

## Methods

**Fig. 1:**
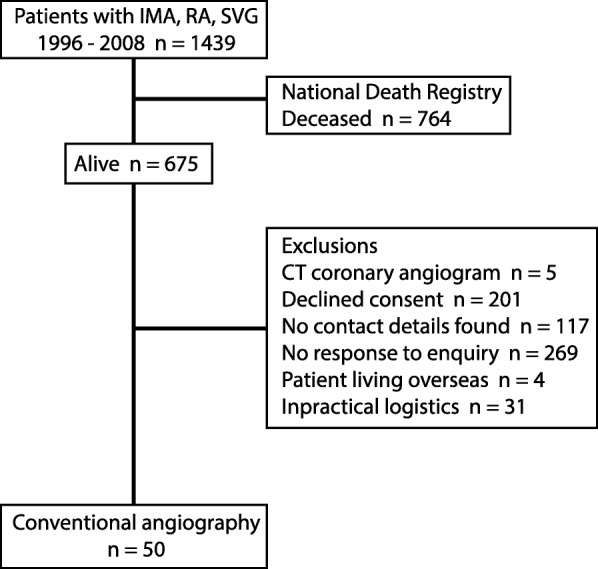
PRISMA flow diagram. A historical cohort up to 19 years postoperative where each patient had internal mammary and radial artery and saphenous vein graft. Research protocol conventional angiography provided high resolution images to detect lumen irregularity

We conducted a prospective observational angiographic study of patients who had received coronary bypass surgery (CABG) using at least one of each of the three conduits, IMA, RA and SVG, and who were *a minimum* of 7 years post-operative, with no upper limit to duration postoperative, Fig. 1. Thus, all postoperative angiograms performed in the early and intermediate periods postoperative were excluded. Conventional angiography was used for optimal accuracy of imaging of conduit lumen irregularity. Participants were identified from the Royal Melbourne Hospital institutional database, and mortality was determined by linkage to the national death registry (Australian Institute Health of Welfare). Postmortem examinations are not routinely performed, and no records were available. All angiography was performed at the Royal Melbourne Hospital, receiving research protocol driven conventional angiography between 2012 to 2017.

### Ethics approval and consent to participate

**Fig. 2:**
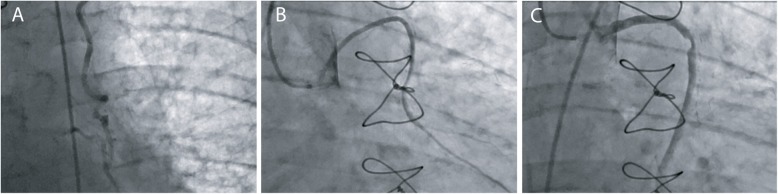
Conduit angiogram examples. **a**, left internal mammary artery, **b**, radial artery, **c**, saphenous vein

The Melbourne Health human ethics committee approved the study and written informed consent was obtained for all participants, HREC 2011.164. Living patients were approached if they had received coronary artery bypass surgery where all three types of conduit (IMA, RA and SVG) were used. Participants who refused conventional angiography, had contraindications to angiography, who underwent CT coronary angiogram, or had angiography for clinical indications, were excluded, Figs. 1 and 2. The cardiologist performing the angiogram and a researcher were the observers and neither were blinded to the grafting strategy. Following angiography, the report was distributed to the patient’s general practitioner and usual treating cardiologist; and the results were discussed directly with the patient at the time of the angiogram. At no time was a therapeutic intervention undertaken or advised at the same time as the research angiogram. The selection of the conduits and the grafting targets were at the sole discretion of the surgeon. However, during the study time frame, it was usual practice at this institution to graft coronary targets with lesion severity of ≥50%; and precise degrees of coronary stenosis and related patency are presented in the Supplement, Table S1. There were no details provided in the operative notes to explain the selected strategy by the surgeon. Institutional policy was for all patients to be discharged from hospital with a lipid lowering medication and low dose aspirin.

### Conduit harvest

During this study time frame, the LIMA was harvested in a pedicled (non-skeletonized) manner, the RA harvested with an open technique and side branches divided with electrocautery between metal clips and with the use of topical and intraluminal 1% papaverine solution and no intravenous systemic vasodilators; and SVG harvested using an open technique with side branches divided between fine ties or metal clips without additional fat being included adjacent to the vein and gentle dilation of the vein using saline.

### Primary analysis: assessment of perfect patency (absence of conduit lumen irregularity)

The lumen was examined for evidence of irregularity in the same manner as for the native coronary artery angiography. Any irregularity (even a minor irregularity) was classified as irregular. Absence of irregularity was classified as normal. The term “Perfectly Patent”, refers to a conduit being both patent and having a normal lumen angiographic appearance.

### Secondary analysis: assessment of patency (patent even in presence of conduit lumen irregularity)

Conduits were classified as patent or occluded. Arterial grafts considered to have a “string sign” (a diffusely narrowed conduit that failed to fill the native coronary artery via the graft injection), were classified as occluded. Sequential anastomoses were considered as separate grafts.

### Statistics

For continuous variables, the means and standard deviations were provided. In order to adjust for patient level effects and other risk factors, generalised linear mixed model analysis (GLMM) was used, with patency and perfect patency as outcomes, patient as the random effect, and type of conduit as the key predictor. The patient level variables included in these models were duration to the research angiogram postoperatively, diabetes, hypertension, hypercholesterolaemia and atrial fibrillation, and graft level variables of preoperative native coronary stenosis and coronary territory. The patient random effect on patency (var = 0.60, se = 0.92) and perfect patency (var = 0.22, se = 0.74) was small.

A sensitivity test was performed where specific exclusions were made including the RA graft known to be calcified at implantation, the patient describing post angiogram symptoms, or for angiograms performed > 10 years postoperative; or patients from all three of these scenarios being excluded, see Supplementary Materials; and all were consistent with the main analyses.

## Results

Of an original cohort of 1439 patients, 675 remained alive at the times of contact, and a further 627 were excluded, with the majority unable to be located or declined consent, Fig. 1. Fifty patients had angiography at age 74.3 ± 7.0 (range 57–90) years. Research protocol driven angiography was performed at 13.1 ± 2.9 (range 7–19) years postoperative. Patients received 3.9 ± 1.0 (range 3–6) anastomoses. Demographic details are listed in Table 1. Six patients had previous postoperative angiography at least 7 years prior to their current research angiogram; one of whom had a drug eluting stent placed to a diseased SVG at 9 years postoperatively; and at research angiography at 16 years postoperatively (7 years post stent), this graft was occluded. One patient denied chest pain symptoms at the time or recruitment; however, after the angiogram the patient admitted to mild atypical chest pain symptoms; and had patent grafts.

**Table 1:** Demographic details *n* = 50

Variable	***n (%)***
Male	48 (96)
Symptoms	1 (2)
Current smoker	2 (4)
Diabetes	21 (42)
Hypertension	37 (74)
Hypercholesterolemia	29 (58)
Family history of IHD	1 (2)
Dialysis	2 (4)
Chronic lung disease	1 (2)
Atrial fibrillation	8 (16)
Coronary stent pre-research angiogram	1 (2)

*IHD* ischaemic heart disease

### Distribution of anastomoses

There were 196 anastomoses which were relatively evenly distributed between conduits IMA *n* = 62, RA *n* = 77 and SVG *n* = 57. The distribution of grafts was biased by IMA predominantly being grafted to the LAD territory, with RA and SVG predominantly elsewhere, *P* < 0.001, Tables 2 and 3. Within the LAD territory, RA and SVG were predominantly grafted to the diagonal arteries. Details of anastomosis distribution according to conduit, coronary stenosis and coronary territory are listed in Supplemental Table S1. Although there were differences shown in the raw data within the comparisons of patency according to the coronary territory, after an adjustment of the conduit to the model, there were no influences on perfect patency, *P* = 0.289 or patency, *P* = 0.997, Table 4. Sequential grafting was mostly with arterial conduits and composite Y-grafting predominantly occurred with RA, Table 2.

**Table 2:** Distribution of anastomoses

	IMA ***n (%)***	RA ***n (%)***	SVG ***n (%)***	Total ***n (%)***
*Coronary Territory*				
LAD	57	8	6	71 (36)
Cx	4	50	18	72 (37)
RCA	1	19	33	53 (27)
Total	62 (32)	77 (39)	57 (29)	196 (100)
*Anastomosis*				
End-Side	53	67	56	176
Sequential	9	10	1	20
*Graft Origin*				
Aorta	6	70	56	132
Pedicled	56	0	0	56
Y graft	0	7	1	8

*LAD* left anterior descending artery territory, *IMA* internal mammary artery, *RA* radial artery, *SVG* saphenous vein graft, pedicled, origin from the subclavian artery, Y graft, composite graft between two conduits

**Table 3:** Distribution of conduits according to coronary branch target

Coronary branch	IMA	RA	SVG	Total
LAD	46	1	1	48
D1	10	5	5	20
D2	1	2	0	3
Intermediate	1	5	3	9
M1	2	21	9	32
M2	0	19	5	24
M3	1	5	1	7
RCA	0	1	1	2
PDA	1	14	26	41
LVBr	0	4	6	10
Total	62	77	57	196

Intermediate artery was grouped with the circumflex territory

*IMA* internal mammary artery, *RA* radial artery, *SVG* saphenous vein graft, *LAD* left anterior descending artery, *D1–2* diagonal arteries, *M1–3* marginal arteries, *RCA* right coronary artery, *PDA* posterior descending artery, *LVBr* left ventricular branch artery

**Table 4:** Multivariable comparison of patency according to coronary territory

Comparison	Perfect Patency ***n*** (%)	P (GLMM)	Patency ***n*** (%)	P (GLMM)
LAD, CX, RCA		0.997		0.289
LAD Cx	63/71 (88.7) 53/72 (73.6)	0.940	66/71 (93.0) 67/72 (93.1)	0.277
LAD RCA	63/71 (88.7) 25/53 (47.2)	0.941	66/71 (93.0) 46/53 (86.8)	0.901
Cx RCA	53/72 (73.6) 25/53 (47.2)	0.997	67/72 (93.1) 46/53 (86.8)	0.136

*P (GLMM) P* value adjusted for patient level effects and other risk factors, *GLMM* generalised linear mixed model analysis (see Methods for variables), *LAD* left anterior descending artery, *Cx* circumflex artery, *RCA* right coronary artery, univariable analysis, see Supplement Table S7

### Global predictors

Conduit type was the only significant predictor for perfect patency, *P* < 0.001. Preoperative native coronary stenosis OR 1.07, 95% CI (1.02, 1.11), *P* = 0.001; duration to the research angiogram OR 0.69, 95 CI (0.51, 0.91), *P* = 0.010, and conduit type *P* = 0.049, were predictors of patency.

### Conduit analysis

Perfect patency was 96.8% for IMA, 92.2% for RA, and 17.5% for SVG indicating that the majority of patent SVG had atheroma present and the arterial grafts were normal. The IMA perfect patency was not significantly different to the RA (absolute difference 4.6%, *P* = 0.265) but was higher than SVG (absolute difference 79.3%, *P* < 0.001). Similarly, RA perfect patency was significantly higher than SVG (absolute difference 74.7%, *P* < 0.001).

**Fig. 3:**
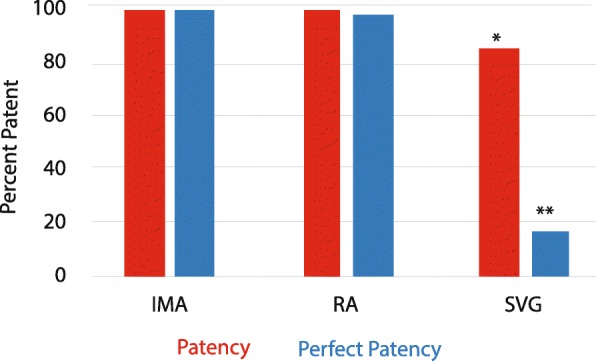
Conduit patency and perfect patency (GLMM) *n* = 196 anastomoses. Arterial conduit patency was high, not different from each other despite differing coronary territory grafting and all but one was also perfectly patent. However, vein graft had lower patency and very low perfect patency with progressive decline over time expected. GLMM, generalized linear mixed model analysis, IMA, internal mammary artery, RA, radial artery, SVG, saphenous vein graft, *, *P* = 0.021 SVG vs. IMA, **, *P* < 0.001 SVG vs. IMA or RA

The incidence of patency and perfect patency for all grafts is shown in Table 5, Supplemental Tables S1 and S2 and Fig. 3. All patent IMA were perfectly patent, and only one patent RA was not perfectly patent. Patency for IMA was 96.8%, RA was 93.5%, and SVG was 82.5%. The IMA patency was not significantly different to the RA (absolute difference 3.3%, *P* = 0.146) but was higher than SVG (absolute difference 14.3%, *P* = 0.016). The RA patency was higher than for SVG (absolute difference 11%), however but was the difference was not significant (*P* = 0.170). When combining all arterial grafts versus SVG, both perfect patency and patency were higher for arterial grafts (perfect patency 94.2% vs. 17.5%, *P* < 0.001 and patency 95% vs. 82.5%, *P* = 0.033).

**Table 5:** Multivariate comparison of patency according to conduit

Comparison	Perfect patency ***n*** (%)	P (GLMM)	Patency ***n*** (%)	P (GLMM)
IMA, RA, SVG		< 0.001		0.049
IMA vs. RA	60/62 (96.8) 71/77 (92.2)	0.309	60/62 (96.8) 72/77 (93.5)	0.169
IMA vs. SVG	60/62 (96.8) 10/57 (17.5)	< 0.001	60/62 (96.8) 47/57 (82.5)	0.021
RA vs. SVG	71/77 (92.2) 10/57 (17.5)	< 0.001	72/77 (93.5) 47/57 (82.5)	0.175
Arterial, SVG		< 0.001		0.037
Arterial vs. SVG	131/139 (94.2) 10/57 (17.5)	< 0.001	132/139 (95.0) 47/57 (82.5)	0.037

*P (GLMM) P* value adjusted for patient level effects and other risk factors, *GLMM* generalised linear mixed model analysis (see Methods for variables), *IMA* internal mammary artery, *RA* radial artery, *SVG* saphenous vein graft, see Supplementary Materials for sensitivity testing, univariable analysis, see Supplement Table S2

**Fig. 4:**
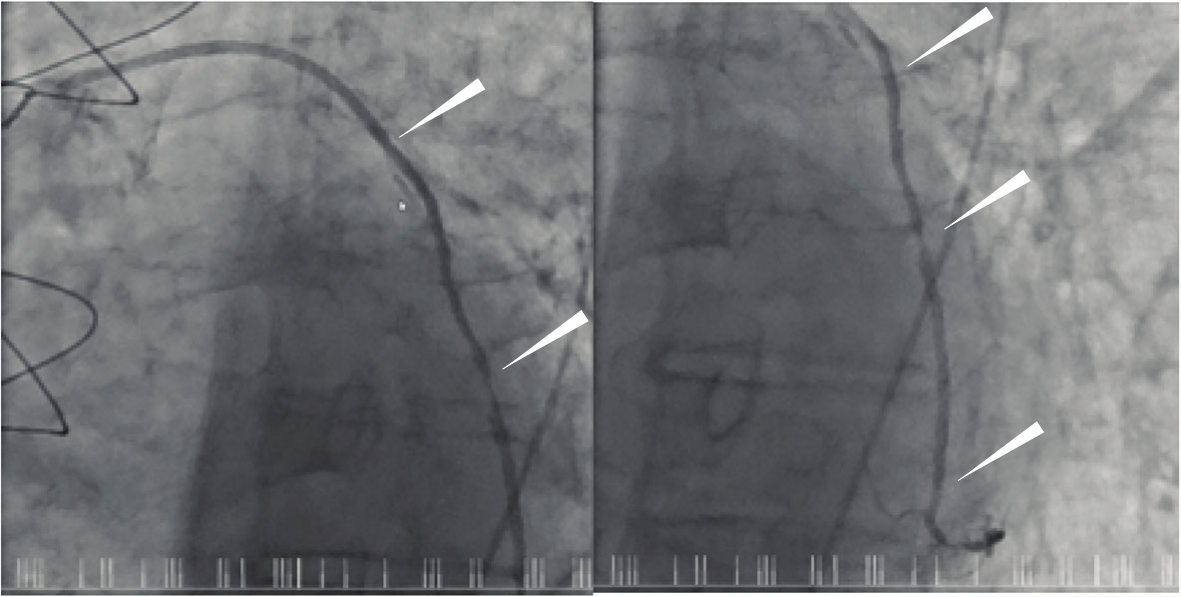
The only non-perfectly patent arterial conduit. The only patent, but diseased arterial conduit was a radial artery to second marginal, illustrated by arrows. However, it was noted to be diseased at the time of surgery 11 years prior and it is not known if there has been any progression postoperatively. The native coronary artery has severe disease and was noted to be diseased at the time of surgery. https://s3.amazonaws.com/igraft/3Vangio/PreopDiseasedRA.mp4. From the operation report: *“The left radial artery was exposed but on harvesting was found to be extensively calcified and not useable. The right radial artery was harvested; this was a 2.2 mm artery with at least moderate medial wall calcification”*

In the case of the single radial artery graft that did exhibit lumen irregularity, the operative report detailed significant calcification of this right RA at the time of the surgery and at late angiography, this RA conduit remained patent with an irregular lumen, Fig. 4, Supplemental Figure S1; but it was not possible to determine if there had been any progression of calcification or atheroma in the postoperative period. Additional sensitivity analyses were therefore conducted, see Supplement Tables S3, S4, S5 and S6, which did not alter the findings.

### Effect of preoperative coronary artery stenosis

The overall effect of the degree of coronary stenosis present at the time of surgery was not significant for perfect patency, *P* = 0.317; but was significant for patency, *P* < 0.001, Table 6. RA and SVG were significantly impacted for patency, but IMA was not. The full distribution of coronary stenosis with conduit type and territory is listed in Supplement Table S1 and conduit distribution according to stenosis grouped ranges Supplement Tables S7 and S8.

**Table 6:** Comparison of conduit patency according to the influence of preoperative coronary stenosis

Variable	Perfect patency P (GLMM)	Patency P (GLMM)
IMA, RA, SVG	0.317	< 0.001
IMA	0.204	0.133
RA	0.030	0.007
SVG	0.275	0.032

*P (GLMM) P* value adjusted for patient level effects and other risk factors, *GLMM* generalised linear mixed model analysis (see Methods for variables), *IMA* internal mammary artery, *RA* radial artery, *SVG* saphenous vein graft

Patients in whom all three conduits were considered perfectly patent (*n* = 6), there were no statistically significant differences with the remainder of patients (*n* = 43), Table 7.

**Table 7:** COPD, chronic obstructive pulmonary disease, AF, atrial fibrillation, PCI, percutaneous coronary intervention, Redo CAGS, reoperation coronary artery bypass surgery

Variable	Patent + normal all 3 conduits (***n*** = 6)	Other (***n*** = 43)	***P*** value
Age	58.8 ± 9	61.2 ± 7.2	0.455
Gender	6 (100)	41 (95.3)	1
**Preoperative factors**			
Current Smoker	0	2 (4.7)	1
Diabetes	4 (66.7)	16 (37.2)	0.21
Hypertension	5 (83.3)	31 (72.1)	1
Family History	0	1 (2.4)	1
Cholesterol	4 (66.7)	24 (55.8)	0.688
Dialysis	0	1 (2.3)	1
COPD	0	3 (7)	1
AF	1 (16.7)	6 (14)	1
**Postoperative factors**			
Angina	0	1 (2.3)	1
Myocardial Infarction	0	0	
PCI	0	2 (4.7)	1
Redo CAGS	0	0	

## Discussion

### High perfect patency in arterial conduits

The most important finding is that arterial conduits that were patent, appeared normal in the late period postoperatively. This is different to SVG which rarely appears normal in the late period, with most (82.5% in this series) having some lumen irregularity consistent with conduit wall atheroma. Although, it is well known for patent LIMA to appear normal irrespective of the duration postoperatively, it has generally been assumed that this would not be true for RA. These data find that when patent, *both* arterial conduits appear angiographically normal (atherosclerosis-free) in the late period. The conclusion to be drawn is that a diseased conduit such as SVG may be expected to have ongoing progressive atheroma formation which ultimately, may cause graft failure by way of hemodynamically significant stenosis or occlusion; whereas the normal arterial conduits may be expected to remain normal indefinitely.

In the single case of a diseased RA, it was noted that there was significant disease present at the time of surgical implantation 11 years prior to angiography, Fig. 4, Supplemental Figure S1. This conduit did not occlude in the interim which was surprising, and the expectation of what may have occurred with SVG. What is not clear is if there was any progression in the severity of the RA disease postoperatively, or alternatively, if the degree of disease remained stable.

### High patency of arterial conduits

By selecting patients in the late period only, we have reduced the probability that a patent SVG would have been recorded in the early or intermediate period, that could have later occluded in the late period. Thus, all patients were from the late period postoperatively. Both conduits had superior perfect patency and patency compared to SVG at a mean of more than 10 years postoperative, Table 5, Fig. 3.

### Coronary territory

The similarity of IMA and RA patencies despite clear grafting preferences for IMA to the LAD territory and RA for the non-LAD territory, is a novel finding. We attempted to reduce bias by the use of research protocol driven rather than symptom indicated angiography and the presence of all three conduits within each patient allowing for elimination of many patient and medication factors between conduits within each individual patient. However, the preferences in grafting strategy did not alter by our approach. Yet, we found no differences according to coronary territory, Table 4. This was surprising as there are many studies that demonstrate highest patency in the LAD and lowest patency in the RCA territories [1, 10]. These data could potentially challenge some conventional wisdoms. The first, LIMA-LAD being unique in some way, is based on the historical considerations of Loop in 1986 [13] . Their analysis considered LIMA (as the only arterial conduit), being applied to the LAD (the only coronary target for LIMA); and all other grafts were SVG. An alternative interpretation of their data is that they observed the survival impact of one arterial graft compared to the exclusive use of venous grafts. Our data suggest an alternative to the conventional view – that all arterial grafts, if they remain patent in the early period – may have long term, perfect patency. With this hypothesis, the similarity of perfect patency according to coronary territory is explained.

RA and SVG were both grafted predominantly to the non-LAD territories and RA perfect patency was higher than SVG, *P* < 0.001, see Supplemental Table S1. Patency was not significant despite an 11% absolute difference, and it is considered likely that a Type II statistical error due to the small sample size was present. Combining both arterial conduits still maintained superiority over SVG, Table 5.

The patency of SVG of 82.5%, was higher than expected from the literature (47–64%) [3–7], for this time period post operatively. The higher patency may have reflected survivor bias, which may have been unrelated to conduit selection. Alternatively, a higher proportional of grafts being arterial conduits being used 139/196 (71%), and with a lower failure rate may have led to improved survival. However, the majority of SVG showed evidence of atherosclerosis with only 17.5% being considered normal. The observed difference between arterial and venous conduits were not due to poor results in the venous group.

### Coronary stenosis

The effect of coronary stenosis was significant for RA for both perfect patency and for patency; and was significant for patency for SVG, Table 6. These findings are expected; although the interaction with coronary territory is a confounder. Examining the individual anastomoses by coronary territory, conduit and degree of coronary stenosis, for anastomoses to coronary stenosis of < 80% were mostly patent for RA and SVG; in contrast to the conventional view that most or all of such anastomoses would fail due to competitive flow, Table 4.

### Study implications

Arterial conduits that did not fail in the early period, showed no evidence of progressive atheroma and so theoretically may never fail; whereas most SVG that do not fail in the early period can still be expected to develop conduit atheroma over time which would be expected to be progressive. We have previously reported a survival advantage to total arterial revascularisation compered to any use of SVG [11]. The absence of progressive disease in arterial grafts may be the mechanism whereby better long-term survival occurs. Further, there may be relatively little difference between IMA and RA grafts, and that arterial grafts should be considered as equivalent grafts from this study, noting considerable bias for IMA use to the LAD territory. We have reported that the LIMA-RA-Y graft configuration provides the same survival advantage as other total arterial revascularisation configurations, and is superior to any use of SVG in the late period after surgery with low donor site morbidity [14, 15].

### Study strengths and limitations

The key strength of this study is that each patient had at least one of the three conduits of interest. Consequently, patients acted as their own controls, with identical patient factors affecting all of the conduits equally; other than for the influence of the specific coronary target to which the conduit was grafted. The second key strength was that the cohort was within the “late” period postoperative period. This mitigates the common failing of many series whereby some of the cohort lie within the “early” or “intermediate” period during which time there could be higher SVG patency that could potentially decrease, as more SVG occlude prior to the “late” period of more than 10 years. Use of conventional angiography allowed optimal examination of the lumen for irregularity as a marker of conduit atherosclerosis; and this enhanced the validity of this part of the analysis in comparison to CT coronary angiography, which was considered to be less accurate.

The key weaknesses of the study are that it is a non-randomised, observational study, included only survivors (which may positively bias patency to be higher than for non-survivors), and excluded patients who had angiography for clinical indications (i.e. restricted to research protocol consenting patients). Whilst the patient, environment and medication factors would be identical between conduits for each individual patient, differences could exist between patients and strict control of all such variables was not possible. Additionally, there was a predominant bias for use of the IMA to the LAD territory; and for the other two conduits for the non-LAD territory and this may potentially negatively bias the patency of SVG and RA compared to IMA. Finally, the sample size was relatively small as many late term survivors were very old and did not want any further investigation.

## Conclusion

All but one patent internal mammary artery or radial artery grafts had perfect patency and had superior perfect patency and overall patency compared to saphenous vein grafts.

## Supplementary information

**Additional file 1: Table S1.** Degree of coronary stenosis at the time of surgery according to conduit type and coronary territory. **Table S2.** Multivariate comparison of patency according to conduit. **Table S3.** Comparison of patency rates excluding patient with known preoperative radial artery disease *n* = 192 anastomoses. **Table S4.** Comparison of patency rates excluding patient with angiogram at 7 years *n* = 193 anastomoses. **Table S5.** Comparison of patency rates excluding patient who did not reveal presence of symptoms until after angiography *n* = 191 anastomoses. **Table S6.** comparison of patency rates with combined exclusion of preoperative RA disease and patient with angiography at 7 years and patient who declared symptoms after angiography *n* = 184 anastomoses. **Table S7.** Multivariable and univariable comparison of patency according to coronary territory. **Table S8.** Distribution of grafts by coronary territory and categories of coronary stenosis. **Figure S1.** The only radial artery with irregular lumen 10.6 years postoperative (which was calcified at the time of surgery).

## Data Availability

The datasets used and/or analysed during the current study are available from the corresponding author on reasonable request.

## Abbreviations

LIMA: Left internal mammary artery
IMA: Internal mammary artery
RA: Radial artery
SVG: Saphenous vein graft
LIMA-RA-Y: Composite graft where RA is sutured to the side of LIMA
Patent: Conduit which conducts blood to the target coronary artery
Perfectly patent: Conduit where conventional angiographic lumen appearance has no irregularity (normal)
CABG: Coronary artery bypass surgery
GLMM: Generalized linear mixed model analysis
