# Supplementary material for: Patency of conduits in patients who received internal mammary artery, radial artery and saphenous vein grafts
Source: BMC Cardiovasc Disord. 2020 Mar 24;20:148. doi: 10.1186/s12872-020-01433-0 (PMC7092416; doi:10.1186/s12872-020-01433-0)
Supplement: Supplementary file 1 — Additional file 1: Table S1. Degree of coronary stenosis at the time of surgery according to conduit type and coronary territory. Table S2. Multivariate comparison of patency according to conduit. Table S3. Comparison of patency rates excluding patient with known preoperative radial artery disease n = 192 anastomoses. Table S4. Comparison of patency rates excluding patient with angiogram at 7 years n = 193 anastomoses. Table S5. Comparison of patency rates excluding patient who did not reveal presence of symptoms until after angiography n = 191 anastomoses. Table S6. comparison of patency rates with combined exclusion of preoperative RA disease and patient with angiography at 7 years and patient who declared symptoms after angiography n = 184 anastomoses. Table S7. Multivariable and univariable comparison of patency according to coronary territory. Table S8. Distribution of grafts by coronary territory and categories of coronary stenosis. Figure S1. The only radial artery with irregular lumen 10.6 years postoperative (which was calcified at the time of surgery). [file 12872_2020_1433_MOESM1_ESM.pdf]

SUPPLEMENTARY MATERIALS

Patency of mammary or radial artery and saphenous vein in patients with all three conduits

Royse, A. et al. 2020

Table S1. Degree of coronary stenosis <u>at the time of surgery</u> according to conduit type and coronary territory

**Stenosis at surgery**	**IMA**			**RA**			**SVG**			**Total**
	n	**Pat**	**PPat**	***n***	**Pat**	**PPat**	***n***	**Pat**	**PPat**	
40	2	2	2	1	0	0	0	0	0	3
50	2	1	1	5	5	5	8	6	2	15
60	4	4	4	4	2	2	4	3	1	12
70	8	8	8	6	5	5	7	5	3	21
80	18	18	18	16	16	16	6	6	0	40
90	18	17	17	20	20	19	12	12	0	50
95	1	1	1	7	7	7	2	1	0	10
99	1	1	1	6	6	6	3	3	0	10
100	8	8	8	12	11	11	15	12	4	35
Overall Total	62	60	60	77	72	71	57	47	10	196
LAD territory										
40	2	2	2	0	0	0	0	0	0	2
50	2	1	1	0	0	0	1	1	1	3
60	4	4	4	2	0	0	1	1	0	7
70	6	6	6	1	1	1	1	0	0	8
80	17	17	17	2	2	2	0	0	0	19
90	18	17	17	2	2	2	2	2	0	22
95	0	0	0	1	1	1	0	0	0	1
99	1	1	1	0	0	0	0	0	0	1
100	7	7	7	0	0	0	1	1	1	8
Total	57	55	55	8	6	6	6	5	2	71
Circumflex territory										
40	0	0	0	1	0	0	0	0	0	1
50	0	0	0	4	4	4	4	2	0	8
60	0	0	0	2	2	2	1	1	1	3
70	2	2	2	2	1	1	2	2	1	6
80	1	1	1	12	12	12	3	2	0	16
90	0	0	0	12	12	11	4	4	0	16

95	0	0	0	4	4	4	1	1	0	5
99	0	0	0	3	3	3	1	1	0	4
100	1	1	1	10	10	10	2	2	0	13
Total	4	4	4	50	48	47	18	15	2	72
Right coronary artery territory										
50	0	0	0	1	1	1	3	3	1	4
60	0	0	0	0	0	0	2	1	0	2
70	0	0	0	3	3	3	4	3	2	7
80	0	0	0	2	2	2	3	3	0	5
90	0	0	0	6	6	6	6	6	0	12
95	1	1	1	2	2	2	1	0	0	4
99	0	0	0	3	3	3	2	2	0	5
100	0	0	0	2	1	1	12	9	3	14
Total	1	1	1	19	18	18	33	27	6	53

Table S2. Multivariate comparison of patency according to conduit

Comparison	Perfect Patency n (%)	P (GLMM)	P (Fisher)	Patency n (%)	P (GLMM)	P (Fisher)
IMA, RA, SVG		<0.001			0.049	
IMA vs. RA	60/62 (96.8)<br>71/77 (92.2)	0.309	0.461	60/62 (96.8)<br>72/77 (93.5)	0.169	0.298
IMA vs. SVG	60/62 (96.8)<br>10/57 (17.5)	<0.001	0.013	60/62 (96.8)<br>47/57 (82.5)	0.021	<0.001
RA vs. SVG	71/77 (92.2)<br>10/57 (17.5)	<0.001	0.055	72/77 (93.5)<br>47/57 (82.5)	0.175	<0.001
Arterial, SVG		<0.001			0.037	
Arterial vs. SVG	131/139 (94.2)<br>10/57 (17.5)	<0.001	0.009	132/139 (95.0)<br>47/57 (82.5)	0.037	<0.001

P (GLMM), P value adjusted for patient level effects and other risk factors, GLMM, generalised linear mixed model analysis (see methods for variables), Fisher, Fisher Exact Test for univariable patency analysis, IMA, internal mammary artery, RA, radial artery, SVG, saphenous vein graft, see supplementary materials for sensitivity testing [relates to Table 5 in the paper]

Sensitivity testing
The full analysis is replicated below according to whether the patient with the known calcified radial artery was excluded from the analysis (Table S1), or analysis restricted to > 10 years postoperative (Table S2), or the patient that did not declare symptoms until after his angiogram (Table S3) or all three removed (Table S4). None of these analyses were meaningfully different to the main analysis.

Table S3. Comparison of patency rates **excluding patient with known preoperative radial artery** disease *n* = 192 anastomoses

**Comparison**	**Patency *n* (%)**	**P (GLMM)**	**P (Fisher's)**	**Perfect Patency *n* (%)**	**P (GLMM)**	**P (Fisher's)**
IMA, RA, SVG		0.043			<0.001	
IMA vs. RA	59/61 (96.7)<br>71/76 (93.4)	0.147	0.461	59/61 (96.7)<br>71/76 (93.4)	0.316	0.461
IMA vs. SVG	59/61 (96.7)<br>45/55 (81.8)	0.017	0.013	59/61 (96.7)<br>10/55 (18.2)	<0.001	<0.001
RA vs. SVG	71/76 (93.4)<br>45/55 (81.8)	0.172	0.052	71/76 (93.4)<br>10/55 (18.2)	<0.001	<0.001
Arterial, SVG		0.017			<0.001	
Arterial vs. SVG	130/137 (94.9)<br>45/55 (81.8)	0.033	0.009	130/137 (94.9)<br>10/55 (18.2)	<0.001	<0.001

GLMM, generalised linear mixed model, IMA, internal mammary artery, RA, radial artery, SVG, saphenous vein graft, Fisher's, Fisher exact test

Table S4. Comparison of patency rates **excluding patient with angiogram at 7 years** *n* = 193 anastomoses

**Comparison**	**Patency *n* (%)**	**P (GLMM)**	**P (Fisher's)**	**Perfect Patency *n* (%)**	**P (GLMM)**	**P (Fisher's)**
IMA, RA, SVG		0.029			<0.001	
IMA vs. RA	59/61 (96.7)<br>71/76 (93.4)	0.146	0.461	59/61 (96.7)<br>70/76 (92.1)	0.265	0.299
IMA vs. SVG	59/61 (96.7)<br>46/56 (82.1)	0.016	0.013	59/61 (96.7)<br>10/56 (17.9)	<0.001	<0.001
RA vs. SVG	71/76 (93.4)<br>46/56 (82.1)	0.175	0.054	70/76 (92.1)<br>10/56 (17.9)	<0.001	<0.001
Arterial, SVG		0.017			<0.001	
Arterial vs. SVG	130/137 (94.9)<br>46/56 (82.1)	0.032	0.009	129/137 (94.2)<br>10/56 (17.9)	<0.001	<0.001

GLMM, generalised linear mixed model, IMA, internal mammary artery, RA, radial artery, SVG, saphenous vein graft, Fisher's, Fisher exact test

Table S5. Comparison of patency rates **excluding patient who did not reveal presence of symptoms until after angiography** *n* = 191 anastomoses

**Comparison**	**Patency *n* (%)**	**P (GLMM)**	**P (Fisher's)**	**Perfect Patency *n* (%)**	**P (GLMM)**	**P (Fisher's)**
IMA, RA, SVG		0.015			<0.001	
IMA vs. RA	58/60 (96.7)<br>70/75 (93.3)	0.146	0.462	58/60 (96.7)<br>69/75 (92.0)	0.253	0.299
IMA vs. SVG	58/60 (96.7)<br>46/56 (82.1)	0.016	0.014	58/60 (96.7)<br>10/56 (17.9)	<0.001	<0.001
RA vs. SVG	70/75 (93.3)<br>46/56 (82.1)	0.175	0.056	69/75 (92.0)<br>10/56 (17.9)	<0.001	<0.001
Arterial, SVG		0.017			<0.001	
Arterial vs. SVG	128/135 (94.8)<br>46/56 (82.1)	0.032	0.010	127/135 (94.1)<br>10/56 (17.9)	<0.001	<0.001

GLMM, generalised linear mixed model, IMA, internal mammary artery, RA, radial artery, SVG, saphenous vein graft, Fisher's, Fisher exact test

Table S6. comparison of patency rates with **combined exclusion of preoperative RA disease and patient with angiography at 7 years and patient who declared symptoms after angiography** *n* = 184 anastomoses

**Comparison**	**Patency *n* (%)**	**P (GLMM)**	**P (Fisher)**	**Perfect Patency *n* (%)**	**P (GLMM)**	**P (Fisher)**
IMA, RA, SVG		0.043			<0.001	
IMA vs. RA	56/58 (96.6)<br>68/73 (93.2)	0.146	0.463	56/58 (96.6)<br>68/73 (93.2)	0.316	0.463
IMA vs. SVG	56/58 (96.6)<br>43/53 (81.1)	0.017	0.013	56/58 (96.6)<br>10/53 (18.9)	<0.001	<0.001
RA vs. SVG	68/73 (93.2)<br>43/53 (81.1)	0.170	0.052	68/73 (93.2)<br>10/53 (18.9)	<0.001	<0.001
Arterial, SVG		0.017			<0.001	
Arterial vs. SVG	124/131 (94.7)<br>43/53 (81.1)	0.033	0.009	124/131 (94.7)<br>10/53 (18.9)	<0.001	<0.001

GLMM, generalised linear mixed model, IMA, internal mammary artery, RA, radial artery, SVG, saphenous vein graft, Fisher's, Fisher exact test

Table S7. Multivariable and univariable comparison of patency according to coronary territory

Comparison	Perfect Patency n (%)	P (GLMM)	P (Fisher)	Patency n (%)	P (GLMM)	P (Fisher)
LAD, CX, RCA		0.997			0.289	
LAD	63/71 (88.7)	0.940	<0.031	66/71 (93.0)	0.277	1.000
Cx	53/72 (73.6)			67/72 (93.1)		
LAD	63/71 (88.7)	0.941	<0.001	66/71 (93.0)	0.901	0.358
RCA	25/53 (47.2)			46/53 (86.8)		
Cx	53/72 (73.6)	0.997	0.003	67/72 (93.1)	0.136	0.357
RCA	25/53 (47.2)			46/53 (86.8)		
LAD+Cx	116/143 (81.1)		<0.001	133/143 (93.0)		0.250
RCA	25/53 (47.2)			46/53 (86.8)		

P (GLMM), P value adjusted for patient level effects and other risk factors, GLMM, generalised linear mixed model analysis (see methods for variables), Fisher, Fisher Exact Test for univariable patency analysis, LAD, left anterior descending artery, Cx, circumflex artery, RCA, right coronary artery [relates to Table 4 in the paper]

Table S8. Distribution of grafts by coronary territory and categories of coronary stenosis.

Coronary Territory	Coronary stenosis				
	<50%	50-70%	70-90%	>90%	Total
LAD	1	4	17	48	70
Cx	2	5	7	57	71
RCA	0	3	3	46	52
Total	3	12	27	151	`93

Figure S1. The only radial artery with irregular lumen 10.6 years postoperative (which was calcified at the time of surgery)

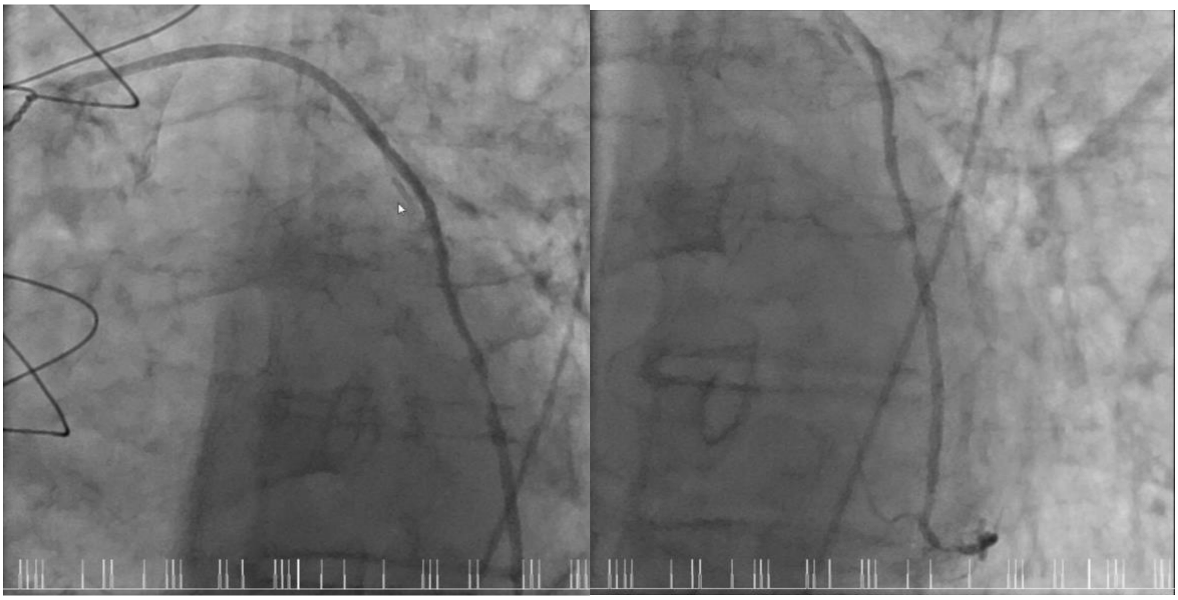

Download video of the angiogram (30 Mb file)
https://s3.amazonaws.com/igraft/3Vangio/PreopDiseasedRA.mp4

From the operation report:

"The left radial artery was exposed but on harvesting was found to be extensively calcified and not useable. The right radial artery was harvested; this was a 2.2 mm artery with at least moderate medial wall calcification."
